# School Age Neurological and Cognitive Outcomes of Fetal Growth Retardation or Small for Gestational Age Birth Weight

**DOI:** 10.3389/fendo.2019.00186

**Published:** 2019-03-28

**Authors:** Brigitte Vollmer, Caroline J. Edmonds

**Affiliations:** ^1^Clinical and Experimental Sciences, Faculty of Medicine, University of Southampton, Southampton, United Kingdom; ^2^Paediatric and Neonatal Neurology, Southampton Children's Hospital, University Hospital Southampton NHS Foundation Trust, Southampton, United Kingdom; ^3^School of Psychology, University of East London, London, United Kingdom

**Keywords:** small for gestational age, fetal growth restriction, cognitive outcome, neuromotor outcome, school age

## Abstract

Children who were growth restricted *in utero* (FGR) and are born small for gestational age (SGA) may experience poorer long term neurological and cognitive outcomes. Those also born preterm may have particular difficulties. The objective of this paper was to review the literature on school age neurocognitive outcome for term and preterm children that was published in the last 15 years. Considering term born children first, there is evidence that these children are at higher risk for Cerebral Palsy (CP) than those born appropriate for gestational age (AGA); information on neuromotor function in the absence of CP is somewhat contradictory. With regards to cognitive outcome, the most common finding was that being born SGA and/or FGR at term does not impact negatively on general intellectual functioning, commonly assessed by IQ scores. There was some indication that they may experience particular problems with attention. With regards to children born preterm, the risk of CP appears not to be increased compared to those preterms born AGA. For preterm children who do not develop CP, motor outcome is more affected by post-natal and post-neonatal brain growth than intrauterine growth. In contrast to term born children, preterm SGA and/or FGR children are at increased risk of cognitive and behavioral difficulties, and in common with term born children, are at higher risk than their AGA counterparts of difficulties with attentional control. In conclusion, preterm born SGA and/or FGR children are at higher risk of neurodevelopmental problems in the school years. It is important to continue to follow up children into the school age years because these difficulties may take time to emerge, and may be more visible in the more demanding school environment.

Low birth weight and poor fetal growth affects a significant proportion of newborns and pregnancies worldwide, and have been associated with a risk for impaired neurodevelopment across multiple domains (1, 2) for both individuals born preterm and born term, with a notion that outcomes for those born preterm are likely to be more complex than in those born term.

In the existing literature on neurodevelopmental outcomes, often, small for gestational age (SGA) and fetal growth restriction (FGR) are used interchangeably, but FGR is not the same as SGA. This makes interpretation and comparison of outcome studies often complicated. Study-specific definitions of FGR, SGA and IUGR are included in the tables.

The definition of small-for gestational age (SGA) by the Royal College of Obstetrics and Gynecology (RCOG; https://www.rcog.org.uk/globalassets/documents/guidelines/gtg_31.pdf) in the UK, and also the American College of Obstetricians and Gynecologists, refers to a newborn with a weight or abdominal circumference at birth at less than the 10th centile, either according to population based growth charts or centiles that take into account factors such as gestational age, sex, ethnicity, and maternal characteristics. Further, this can be divided into normal (i.e., constitutionally small), non-placenta mediated growth restriction (for example, chromosomal abnormalities, syndromes, infections), and placental mediated growth restriction. Fetal growth restriction implies that the fetus cannot achieve its growth potential, and this is often indicated by abnormal Doppler studies. Over half of those who are SGA have appropriate fetal growth (“constitutional” SGA), although the likelihood of also having FGR is higher in SGA. Fetal growth restriction can be classified into symmetrical (both body weight and head circumference are affected) and asymmetrical (body weight and/or length may be affected, head size is normal) and according to the time of onset of FGR (early, i.e., before 28 weeks of gestation, late, i.e., after 28 weeks of gestation) (3). Time of onset of FGR is an important factor since early and late onset FGR are distinct phenotypes in terms of placental dysfunction and effects on the brain. Early and late onset FGR affect the brain at different developmental stages and it is therefore likely that different brain regions are affected in a different way, which may partly explain the different outcomes in these two groups (4). Fetal growth retardation and preterm birth are often associated (5). Preterm birth *per se* poses a risk for long term neurological and developmental impairment and the combination with FGR is likely to add to this risk, however, it may be difficult to disentangle the effects of FGR from the effect of prematurity, and, indeed there have been somewhat inconsistent and contradictory finding in existing studies.

Studies have reported differences for the above described groups in neurodevelopmental outcomes, generally reporting favorable outcomes for those with constitutional SGA, whereas those with placenta-mediated FGR appear to be at risk for cognitive and/or behavioral problems later in life (6). In addition, within the FGR group, differences in outcomes are reported between those with symmetrical and those with asymmetrical FGR (7), although, again, some contradictory study results exist. Overall, existing studies are heterogeneous, for example, with regards to definition of growth restriction and small for gestational age, time of onset of FGR, inclusion and exclusion criteria, outcome measures, all of which makes comparison between studies difficult.

Nevertheless, some common themes emerge and in this narrative literature review we will summarize evidence on neuromotor and cognitive long term outcomes of SGA at birth and after FGR. The focus will be on school age outcomes rather than earlier outcomes as toddler age or adult age outcomes, and this will be described separately for children born at term age and for those born preterm. We considered literature published in the last 15 years. We have excluded studies that included children with underlying chromosomal abnormalities or teratogenic exposure.

## Measures Used in Studies on SGA/ FGR Outcomes

While the use of standardized assessments makes cross-study comparison possible, some of the assessments used were more individual and depended on the study location and specific sample. For example, some studies used information from military conscription (8), national registers of learning disability (9), and/or and information extracted from medical records (10).

Neuromotor and neurosensory outcomes are described in some, but not all, studies. In the studies that did describe neuromotor outcomes, only seldom a standardized approach was applied for neurological examination. Diagnosis of Cerebral Palsy (CP) was either based on direct neurological examination assessing posture, movements, muscle tone, and reflexes, or on information extracted from medical notes. In a few studies, and when diagnosis was based on examination, the Surveillance of Cerebral Palsy in Europe (SCPE) criteria for CP were used (11). Functional impairment in those with CP was not always defined, but in some studies the Gross Motor Function Classification System (GMFCS, (12) was used for overall motor function classification. In one study, independently of a diagnosis of CP, gross motor function was assessed using the GMFCS, and hand function using the Manual Ability Classification System (13). The most commonly used tool for assessment of motor skills was the Movement ABC for Children (14), a standardized tool, which assesses dexterity, balance skills, and ball skills. Another standardized test, the Peabody Developmental Motor Scales (15), testing reflexes, stationary, locomotion, object manipulation, grasping, and visual-motor integration, has also been used.

Very rarely are visual function and hearing function described specifically, and, if so, this information was mainly extracted from medical records and reported as a binary outcome (impairment/no impairment).

For outcome measures used to assess general cognitive abilities and specific cognitive functions, there was a large and heterogeneous range of assessments reported, but most of the outcome measures used were standardized neuropsychological tests and questionnaires. For assessing general intellectual functioning, the most frequently used assessment was the Wechsler Intelligence Scale set, most commonly the Wechsler Intelligence Scales for Children (WISC) (16), but also the Wechsler Abbreviated Scales of Intelligence (WASI) (17), and the Wechsler Preschool and Primary Scale of Intelligence (WPPSI) (18). The Wechsler Scales assess performance across cognitive domains and usually include an overall IQ score, and performance and verbal IQ scores. General intellectual functioning was also assessed using the Kaufman ABC (19). More focused neuropsychological assessments were used to examine specific aspects of performance, including attention (the Test of Everyday Attention in Children—TEA-Ch) (20), memory (Rey's Auditory Verbal Learning Test—RAVALT) (21), and design copying of the Rey Ostereith figure (22). Executive functioning was assessed using the NEPSY (23), and parental ratings of children's behavior were also employed. Children's executive functioning at home and/or at school were assessed using the Behavior Rating Inventory of Executive Function (BRIEF) (24). The Strengths and Difficulties Questionnaire (SDQ) (25), a brief behavior screening questionnaire, has parents and/or teachers rate children on emotional symptoms, conduct problems, hyperactivity/inattention, peer relationship problems, and prosocial behavior.

The development of typical and atypical social behavior was assessed using the Social Responsiveness Scale (SRS) (26), and screeners for autism included the Social Communication Questionnaire (SCQ) (27) and Autism Diagnostic Observation Schedule (ADOS) (28).

## School Age Outcomes of SGA and/or FGR in Children Born at Term

The study of neurocognitive outcome after fetal growth restriction in term infants is not subject to the same confounding factors as that in preterm infants because many of the mechanisms that affect preterm birth do not affect term infants. Nevertheless, with studies using different indicators of growth restriction, such as strict SGA criteria or birth weight as a continuum, it can be difficult to make cross study comparisons. Table 1 reports study details from studies that examined school age neurocognitive outcome of SGA and/or FGR in the past 15 years. Of these, most studies defined SGA as birth weight <10th centile (9, 10, 32, 37–40, 46, 48, 49). Two studies assessed growth in head circumference from the prenatal period and/or birth to later childhood (34, 36). Three studies defined FGR in relation to birth weight standard deviation scores (8, 44, 48). Thus, there is disparity with the definition of SGA and FGR across studies that could affect the results. Only 2 studies included information on placental insufficiency, which would allow FGR to be identified rather than babies being SGA (38, 40). Furthermore, some of the studies of term born children also include a proportion of preterm babies (8, 40, 44).

**Table 1:** Included studies for term born children.

**References**	**Study type**	**Definition of fetal growth**	**Participants**	**Neurology/Neuromotor function**	**Cognitive/behavior Outcome measure**	**Cognitive/behavior findings**	**Conclusion**
Beukers (29)	Prospective follow up study comparing term FGR and term AGA children	BWt ratio <10th centile; BWR calculated as BW/expected weight for GA, using the Gardosi customized fetal growth chart 50th centile values (30)	96 term children with FGR; 32 term and birthweight ≥ 2,500 g BY 200–2003 Age at assessment: 12 y	Not assessed	*Child assessment:* IQ–Wechsler Intelligence Scale for Children (WISC). Executive Functioning– Impulse control (stop task); Verbal working memory (WISC Digit span); Visual working memory (Spatial Temporal Span of the Amsterdam Neuropsychological Tasks–ANT); set shifting (Shifting visual set–ANT); planning (Tower of London). *Parent ratings:-* Behavior Rating Inventory of Executive Functions Dutch version (BRIEF)	No difference in IQ, executive functioning, attention or parent rating of EF between FGR and controls. Parent report FGR children more social and attention problems	Neurocognitive outcomes including IQ, executive function, memory and attention comparable in FGR children AGA controls Parent ratings suggested elevated levels of social and attention problems
Emond (31)	Population based cohort, north east Brazil	Low BW: BW 1,500–2,499 g (*n =* 202) Appropriate BW: BW 3,000–3,499 g (*n =* 212); matched for sex and month of birth	Born ≥37 wGA BY 1993–1994 Age at assessment: 8 y 40% of original cohort assessed (*n =* 83 low birth weight; *n =* 81 appropriate birth weight) Analyses adjusted for socio-economic background variables Additional analyses performed to examine head size at birth, age 6 m and 8 y with IQ at age 8 y	Neurological status (muscle tone, focal neurological signs) M-ABC for neuromotor function *Findings:* No significant difference in frequency of focal neurological signs	WISC-III for Full Scale IQ, Performance IQ, Verbal IQ Memory (auditory and verbal) assessed with WISC-III subtests TeaCH for attention abilities SDQ for behavior	After controlling for socio-economic background no significant difference between BW groups for IQ scores, memory function Significant differences between BW groups for dynamic balance skills and eye-hand coordination, selective attention; peer relationships Head size at birth, age 6 m and 8 y significantly associated with IQ age 8 y	Low birth weight not associated with general cognitive abilities, except selective attention. Neuromotor function poorer in low birth weight children. Post-natal head growth more important than birth weight.
Fattal-Valevski (32)	Prospective follow-up study	BW <10th centile for GA according to Israeli birth weight curves (33); Growth data (weight, height, head circumference) from 1 to 2 y, 2–6 y, and 6–9 y	136 IUGR children BY 1992–2002 Age at assessment: Annual follow-up from birth; current assessment 9–10 y	Neurodevelopmental examination score including soft neurological signs. Score expressed as percentage of optimal items out of the total.	Short term memory, coordination skills; IQ assessed by the Wechsler Intelligence Scale for Children—revised (WISC-R)	BW, BL and GA positively correlated with 9–10 y neurodevelopmental score BW, BL, but not GA, positively correlated with 9–10 y IQ scores Better outcome for those with asymmetric IUGR than symmetric	Correlation of IQ with BW, BL but not GA suggests that there is better outcome for IUGR children delivered early Correlation of GA with neurodevelopmental score, suggests prematurity affects other areas of development
Gale (34)	Prospective cohort study	HC growth–expressed as Standard Deviation Score (SDS), measured at 18 weeks gestation and birth, and 9 y, using the British 1990 growth reference data (35)	221 children assessed at 18 weeks gestation, births, 9 months and 9 years BY 1992–1994 Age at assessment: 9 y	Not assessed	Wechsler Abbreviated Scales of Intelligence (WASI)—Full Scale IQ (FSIQ), Verbal IQ (VIQ) and Performance IQ (PIQ)	No significant associations between head circumference at 18 weeks gestation or birth and 9 years old IQ scores. Post-natal head group was associated with IQ. After adjusting for sex, FSIQ, VIQ and PIQ increased with each SD increase in head circumference up to 9 months, and to 9 years. The highest FSIQ were observed in children who had large increases in HC between birth and 9 months, and a further increase between 9 months and 9 years. This was not linked to maternal or home characteristics other than maternal education	Post-natal brain growth is more important than prenatal brain growth for IQ Post-natal head growth greater in children of mothers educated to degree level, or of higher SES
Gale (36)	Prospective cohort study – the Avon Longitudinal Study of Parents and Children cohort (ALSPAC). The cohort studied is the Children in Focus cohort – a random 10% selected from ALSPAC.	HC growth - measured at birth, 4, 8, 12, 25, 31, 37, 43, 49, 61, and 96 months. HC in analyses –birth, 1, 4, and 8 years—expressed as z scores obtained from the study group. Growth between time points calculated	633 children BY 1991–1992 Age at assessment: 8 y	Not assessed	IQ assessed by Wechsler Intelligence Scale for Children (WISC) at 8 years	Children with larger HC had higher IQ scores For each 1 SD increase in HC– after adjusting for gender, GA, parental factors—FSIQ, VIQ and PIQ increased age 8 years IQ at 8 years was associated with HG during infancy	Above average head growth during infancy associated with above average IQ scores at 8 years Post-natal brain growth during infancy more important to later IQ than growth at other times Brain growth after infancy may not compensate for slower growth in the first year of life
Geva (37)	Population based prospective follow up study of single center (Tel Aviv, Israel)	BW <10th centile for GA according to Israeli birth weight curves (33), those with genetic disorders and unrelated co-morbidities excluded	110 IUGR children born at term, 63 control AGA children BY 1992–1995 Age at assessment: 9 y	Not assessed	Visual aural digit span test for digit span—aural-oral, visual-oral, aural-written, visual-written; RAVLT (superspan list learning test); Rey Osterreith Complex Figure test (ROYCF)	Learning functions over trials were similar for IUGR and controls on RAVLT. IUGR children performed more poorly than controls on ROYCF. IUGR children worse performance on immediate digit span than controls. Performance on digit span output mode did not differ between IUGR and control. IUGR children have memory problems particularly when the input is aural.	Memory difficulties in children born IUGR in the short term, not observed in later stages of learning (i.e., processing, consolidation and retrieval not affected) Difficulties when recalling both verbal and complex visuo-spatial material
Geva (38)	Population based prospective follow up study of single center (Tel Aviv, Israel)	BW <10th centile for GA according to Israeli birth weight curves (33), those with genetic disorders and unrelated co-morbidities excluded	138 IUGR children born at term, 64 control AGA children BY 1992–1995 Age at assessment: 9 y	Not assessed	VADS Digit span–aural-oral, visual-oral, aural-written, visual-written; IQ assessed by Wechsler Intelligence Scale for Children–revised (WISC-R)	IQ significantly lower in IUGR group. IUGR children performed worse than controls on verbal STM tasks.	Short term memory difficulties in IUGR children are particularly observed when material presented aurally and oral response required General cognitive performance also poorer in IUGR children relative to controls
Lawlor (39)	Single center, population based prospective birth cohort -Aberdeen Children of the 1950s cohort.	IUG rate measured as BW standardized for gender and GA. SGA calculated as BW < 10th centile, using a customized birthweight-for-gestation standard for the whole cohort	9,792 singleton children and 1,645 sibling pairs BY 1950–1956 Age at assessment: 7, 9 and 11 y	Not assessed	General ability measured by the Moray House picture test (MHPT)	Positive linear association between BW and MHPT, even when adjusted for SES, Maternal height and age, gender and GA. Within sibling comparison of SGA vs. AGA showed no difference in MHPT scores. If AGE compared to SGA outside of sibling pairs, AGA performed more poorly	While BW correlates with general cognitive ability within-sibling analyses suggest that associations between AGA status and cognitive ability in singletons is explained by within family factors, including SES, parental education and intelligence, genetic factors and fixed maternal factors
Leitner (40)	Prospective birth cohort	BW < 10th centile for gestational age, according to the Israeli percentile curves published by Lieberman et al. (33). Late onset IUGR (mid 2nd to 3rd trimester; verified by fetal ultrasound)	123 children with IUGR and 63 AGA controls, 30% of the sample were preterm BY 1992–2002 Age at assessment: 9–10 y Children with CP or severe neurological deficits were excluded	Neurological examination from birth, follow-up and at 9–10 years. 72 item examination. At age 9 years assessment of co-ordination skills, tone, timed performance, graphomotor skills. *Findings:* IUGR group poorer performance in coordination, timed performance, graphomotor skills; lower muscle tone	IQ assessed by Wechsler Intelligence Scale for Children (WISC), School achievement by Kauffman Assessment Battery for Children (K-ABC)	IUGR children performed more poorly than AGA controls on IQ and school achievement, where learning difficulties, particularly language-based difficulties were observed. Attention span, speech and language abilities were poorer in the IUGR group. Subgroup analyses compared IUGR children whose height, weight and head circumference were above (“optimal”) or below (“suboptimal”) the 10th growth percentile at age 9–10 years–the “suboptimal” catch up group performed worse in neurodevelopmental and cognitive tests than “optimal” group	Children with IUGR perform more poorly than their AGA peers on tests of IQ, school achievement, and neurological examination. Early growth ameliorates these group differences
Leonard (9)	Population-based record linkage study of children in Western Australia born 1983–1992 with intellectual disability of unknown cause	Percentage of optimal birthweight (POBW) categorized as %–POBW <85% was equivalent to <10th centile of optimal BW based on data from the 1998–2002 Western Australia birth cohort (35)	2,865 children with intellectual disability, including both term and preterm children BY 1983–1992	Not assessed	Registrations with the Disability Services Commission or educational services defined as “mild to moderate” (85.9%), “severe or profound” (7.4%), Autism spectrum disorder (6.7%)	In infants born at term, less than optimal fetal growth (POBW < 85%) was associated with “mild to moderate” intellectual disability, POBW < 75% was associated with “severe” intellectual disability. These were after adjusting for factors including SES and paternal occupation	Less than optimal uterine growth associated with intellectual disability in term born children. This persists after adjusting for sociodemographic factors. Severe growth restriction in term children associated with severe intellectual disability
Lundgren (8)	Population based cohort study of singleton male infants born from 1973 to 1976 and alive aged 18 years	“Light for gestational age” defined as > −2 SDs from mean BW for GA, AGA defined as between −2 and +2 SDS, “heavy for GA” defined as > +2 SDs from mean BW according to Swedish birth standards (41)	168,068 males conscripted for national service BW 1973–1976 Age at assessment: 18–25 y This study includes term and preterm adults	Not assessed	General intellectual performance assessed on four dimensions, logical/inductive, verbal, spatial and theoretical/technical	Being born SGA associated with increased risk of subnormal performance on all outcome measures compared to those born AGA SGA and small HC conferred additional risk of subnormal logical performance, SGA and short adult stature associated with a 40–50% increased risk in all outcome measures	Being born SGA associated with increased risk of impairment on logical/inductive, verbal, spatial and theoretical/technical dimensions of intellectual performance
Sommerfelt (42)	Multicenter study on causes and consequences of *in utero* growth retardation, the NICHD Study of Successive Small-for-Gestational Age Births (NSSSAB) 3 geographical Scandinavian regions; mothers recruited before 20 wGA	SGA = BW were <15th centile for gestation according to reference standards from the Norwegian Birth Registry (43); above 15th centile AGA	321 SGA and 312 AGA (78% of eligible children) Recruitment <20 w GA between January 1986 and March 1988 Age at assessment: 5 y	PDMS for balance, eye-hand coordination, locomotor abilities., Grooved Pegboard test for manual dexterity *Findings:* Motor problems more frequent in SGA group but no significance group difference	WPPSI for IQ scores (Verbal IQ, Performance IQ) Norwegian version of the Illinois Test of Psycholinguistic Abilities for receptive language and short-term memory	No significant difference in IQ scores, memory tests SGA with poorer motor performance lower scores on tests of visuospatial, manual dexterity, and verbal function	SGA group differs little from AGA peers on neuropsychologic profile and neuromotor outcome SGA group weakness in visuo-spatial skills and manual dexterity
Tanis (44)	Population based regional cohort—subgroup (Longitudinal Preterm Outcome Project study), Netherlands	BW < −1 SD and BW > −1 SD according to GA; Dutch Kloosterman curve (45). Cut-off for SGA (> 1 SD, below the 16th percentile) according to Etude Epidemiologique sur les Petits Ages Gestationnels study	42 SGA children (10 full term) and 336 AGA children (120 full term), Analyses combine MPT and term –GA range is 31–41. Both SGA and AGA groups consisted of mostly preterm children BY 2002–2003 Age at assessment: 7 y	Motor outcomes assessed by Movement Assessment Battery for Children (M-ABC) *Findings:* No significant difference in motor skills between AGA and SGA group	Full Scale IQ scores (FSIQ) from the Wechsler Intelligence Scale for Children (WISC). Selective attention and Attention control assessed by the Test of Everyday Attention in Children (TEA-Ch). Verbal memory assessed using Rey's Auditory Verbal Learning Test (RAVLT). Visuomotor integration assessed by Neuropsychological Assessment. Executive functioning in daily life assessed by the Behavior Rating Inventory of Executive Function (BRIEF)	No differences between AGA and SGA children on FSIQ, attention, verbal memory, visuomotor integration, executive functioning. SGA children 4 times more likely to be in the abnormal range on the Attention Control measure than AGA children	SGA and AGA children similar on IQ, verbal memory, executive functioning and movement tests, but SGA children more likely to be in the abnormal range on attention control
Theodore (46)	Case-control follow up study	SGA defined as BW < 10th centile for GA for New Zealand standards (47)	241 SGA children and 348 aGA controls BY 1995–1996 Age at assessment: 7 y	Not assessed	Full Scale IQ scores (FSIQ) from the Wechsler Intelligence Scale for Children (WISC)	No significant difference in FSIQ between SGA and AGA children. In the whole sample, parental education and birth order were associated with FSIQ	No long term negative effect of being born SGA on FSIQ scores

*AGA, appropriate for gestational age; BW, birth weight; BL, Birth Length; BY-birth year; FGR, fetal growth restriction; GA, gestational age; HC, Head Circumference; IUGR, intrauterine growth restriction; POBW, Percentage of optimal birthweight; SES, socio-economic status; SGA, small for gestational age; w, week; y, year. ANT, Amsterdam Neuropsychological Tasks; CP, Cerebral Palsy; BRIEF, Behavior Rating Inventory of Executive Function; K-ABC, Kaufmann Assessment Battery for Children; M-ABC, Movement ABC for Children; MHPT-Moray House Picture Test; NEPSY, A Developmental NEuroPSYchological Assessment; RAVLT, Rey's Auditory Verbal Learning Test; ROYCF-Rey Osterreith Complex Figure test; PDMS, Peabody Developmental Motor Scales; SDQ, Strengths and Difficulties Questionnaire; STM, short term memory; TeaCH, Test of Everyday Attention in Children; VADS, Visual aural digit span test; WASI, Wechsler Abbreviated Scales of Intelligence; WISC, Wechsler Intelligence Scales for Children; WISC-R, Wechsler Intelligence Scale for Children – revised; WPPSI, Wechsler Preschool and Primary Scale of Intelligence; WRAT, Wide Range Achievement Test. IQ, Intelligence Quotient Score; FSIQ, Full Scale IQ Score; PIQ, Performance IQ Score; VIQ, Verbal IQ score. The terms SGA, IUGR, FGR were used as in the study that is described*.

### Neuromotor Outcomes

Studies assessing only children with CP are reported here and not included in the table (10, 48, 49). An association between Cerebral Palsy (CP) and having been born SGA and/or FGR has been reported for children born at term in outcome studies on SGA and/or FGR, and data from Cerebral Palsy registers support this association (11, 50, 51). Whether FGR leads to CP or whether abnormal brain development that causes CP leads to FGR is debated.

The Surveillance of Cerebral Palsy in Europe (11) group report an increased risk of CP, across the gestational age range, for those born below the 10th centile and above the 97th centile [using the North of England standard (52) and fetal growth standard by Maršál et al. (53)]. Data from the Canadian Cerebral Palsy Registry (10) and from other reports based on CP registry data (49, 50, 54) show that those with CP who were born term and SGA compared to those with CP born AGA had more frequently intrauterine infections, small head circumference at birth, maternal gestational hypertension, placental abnormalities, perinatal asphyxia, and delivery by emergency cesarean section, amongst other risk factors, such as birth defects (congenital microcephaly, teratogenic, genetic, and syndromal) as reported by Blair et al. (48), for the Australian CP register cohort. This illustrates that it has to be kept in mind when interpreting findings on associations between SGA and/or FGR with CP that SGA populations in CP registers tend to include a heterogeneous group of SGA children, i.e., also children who had other risk factors for SGA and/or FGR than placenta-mediated FGR.

Many of the prospective follow-up studies examining neurodevelopmental outcomes of SGA and/or FGR at term excluded children with CP or other co-morbidities, and of those that did not exclude them, few reported on the presence or absence of CP, or neurological signs in the absence of CP. Leitner et al. (40) compared the occurrence of neurological signs in the absence of CP in children who were born with BW <10th centile with a group of children born AGA at age 9 years; all SGA children had onset of FGR in the mid-second to third trimester, verified by fetal ultrasound, and all had asymmetric FGR. They found a significant difference in the quality of neurology between those born after FGR and those born AGA, with poorer scores for to motor coordination, timed coordination performance, grapho-motor skills and lower muscle tone in the FGR group. Similarly, Emond et al. (31), who used the M-ABC at age 8 years found, after controlling for socio-economic variables, significantly lower scores for dynamic balance and eye–hand coordination in those born SGA (<10th BW centile) but similar performance to those born AGA for manual dexterity. Tanis et al. (44), in contrast, did not find a significant difference between a group of children born at term and SGA and a group born AGA at the age of 7 years when assessed with the M-BAC (dexterity, balance, ball skills) and on tests of visuo-motor integration. The findings by Tanis et al are consistent with those by Sommerfelt et al. (42), in 5 years old children born <15th centile (Norwegian Birth Registry standards), who did not find significantly poorer performance in the SGA group when compared to the AGA group on testing with the M-ABC.

### Cognitive and Behavioral Outcomes

In the reviewed studies, many different measures of neurocognitive outcome were employed, which can lead to difficulties summarizing results across studies. With this in mind, the first outcome to be discussed is that which was most commonly used across the reviewed studies—general intellectual functioning. IQ scores acquired from the Wechsler tests were the most commonly reported general cognitive outcome measures (29, 31, 32, 34, 36, 40, 42, 44, 46). There was some cross-study consensus about the effect of fetal growth restriction at term on Wechsler derived IQ scores, with three studies reporting that term FGR children's IQ scores were no different from those of control children (29, 42, 44, 46). In contrast, one study reported that FGR children's IQ scores were significantly lower than controls (40), but it should be noted that this study included both term and preterm children and thus this finding may not be isolated to term FGR children. One study found significant positive correlations between IQ scores and birth weight (32). A further two studies used head circumference at birth as an indication of fetal growth restriction (34, 36) and found that, for optimal IQ scores in later childhood, post-natal head growth in term born children was more important than prenatal head growth (34). In addition, the amount of post-natal head growth in term born children was greater for children whose mothers had been educated to degree level, or whose families had higher SES, which could be a consequence of cognitive stimulation and could also be linked to better nutrition (34). General intellectual functioning was also assessed by standardized measures other than the Wechsler range of tests, including the Kauffman ABC (40) and the Moray House picture test (39). Term children with FGR showed weaker performance than controls on both these assessments. In addition, higher levels of learning difficulties were reported, particularly language-based difficulties, and these were reflected by parental report of increased incidence of special educational needs interventions, particularly remedial education, neurological follow up, speech therapy and psychological intervention (40). However, this sample included 30% preterm children. While scores on the Moray House picture test were not affected by AGA status, birth weight as a continuous variable was associated with scores (39).

Taken together, the most common finding is that being born SGA at term does not impact significantly negatively on general intellectual functioning. The majority of studies that specifically examine FGR term children report similar performance on IQ-type measures as those found in controls (29, 39, 44, 46). Two studies reported that FGR term children have lower general intellectual functioning than controls (39, 40), but one of these studies also included a minority of preterm children, whose presence could account for these results (40). The other of these two studies (39) reported on children with late onset IUGR (occurring in the second to third trimester), thus it is possible that differences in the timing of growth restriction across studies impacts on the reported outcome; as not all studies reported the time at which growth restriction occurred, this interpretation is speculative. Birth weight correlates with later IQ scores in some studies (32), but not all (31), but other studies have reported that this occurs even in the normal birth weight range and therefore is not specific to FGR children (55).

As well as assessing performance on tasks measuring general intellectual functioning, the reviewed studies considered the effect of being born SGA at term on a range of more specific cognitive processes. However, as there was little consensus in either the aspects of cognition examined or the tests used, it is not possible to draw strong conclusions about specific outcomes, but any trends in the literature are considered here. Three studies examined memory processes (29, 38, 44). There was no difference between FGR term children and AGA term controls on a standard, orally presented digit span test or visual working memory (29), or differences between SGA and AGA children on a verbal list learning task (RAVLT) (44), although it should be noted that this latter study included both preterm and term SGA children. When performance on a digit span test that systematically varied both the presentation and recall of numbers was examined, the performance of FGR children was characterized by particular difficulty when the presentation was aural and children were required to make an oral response (38). While these findings require corroboration, they could have implications for classroom teachers as they suggest it might be more useful for children with FGR ifs teaching materials were non-aural (e.g., written/picture material) and children's responses should not always require them to speak aloud, but perhaps make written responses instead.

Two studies explored whether term born SGA children experience difficulties with executive functions, which describes a range of cognitive functions whose neural correlate lies in the frontal lobes and are involved with cognitive control of behavior (56). Executive functions are assessed both by performance on neurodevelopmental assessments, and also by questionnaire based rating scales, often completed by parents, which given an indication of how poor executive control can affect children's daily lives. The evidence on the effects of FGR on term born children's executive functioning was limited and mixed. For example, no differences in performance between FRG and AGA controls were found on a measure of impulse control (stop task) (29), and no differences between term SGA or term AGA on the parent rating form the BRIEF (44). However, SGA children were four times more likely to be in the abnormal range on TEA-Ch Attention Control compared to AGA children (44), although it should be noted that this sample included children born at term or preterm. Even in term born children without growth restriction, head circumference at birth and head circumference to length ratio at birth were predictive of ADHD symptoms at age 5 to 6 years (57). Term born low birth weight children had particular problems with attention (31). Thus, there is limited evidence in terms of the number of studies and within these studies, there is no reliable pattern of results on executive functions in term born FGR children. There were other measures assessed in individual studies only, with no cross-study comparison possible. For example, the presence of communication problems identified from children's medical charts did not differ between AGA and SGA term children who had CP (10).

Some of these studies of term born SGA children highlight the period of catch up growth that is particularly important for long term neurocognitive outcome. General intellectual functioning in childhood is influenced by early brain growth–indexed by growth in head circumference–that occurs in infancy. For example, IQ score at 8 years was associated with greater than expected head growth during infancy, rather than head circumference at birth (31, 36), catch up growth at 2 years predicted 9–10 years IQ scores (40). Later growth is also important for IQ; children with optimal somatic catch-up growth at age 9–10 years, but not 2 years (defined as >10th centile in height, weight and head circumference) had significantly higher scores on neurodevelopmental and cognitive tests at 9–10 years than children with suboptimal catch-up growth (defined as <10th centile in height, weight or head circumference) (40). However, there seem to be better outcomes for term born children with asymmetric FGR, which favors head growth over the rest of the body, compared to children with symmetric FGR (32); this suggests sparing of the head, a neuroadaptive modification to conserve the developing brain.

## School Age Outcomes of SGA and/or FGR in the Context of Preterm Birth

Neurodevelopmental outcome of SGA and/or FGR in preterm (born <37 w GA; very preterm = born <32 w GA; extremely preterm ≤28 wGA at birth) infants is difficult to study since there it is complicated separate the effects of SGA/FGR from the consequences of preterm birth *per se*. The majority of studies investigating school age outcomes of SGA or/and FGR in preterm born children have excluded children with congenital abnormalities, congenital infections, chromosomal abnormalities, or syndromes. However, only in some studies detailed information on antenatal growth is available, and seldom are SGA and FGR clearly distinguished and examined separately; some studies may include constitutionally “small at birth” children. Definitions of SGA and growth references used differ between studies. All this is likely to affect study findings and needs to be kept in mind when interpreting and comparing results from different studies.

Table 2 provides study details from 10 studies on neurocognitive outcomes in preterm school aged children, the majority of which are population based studies that examined outcomes of FGR or SGA published in the past 15 years.

**Table 2:** Included studies for preterm born children.

**References**	**Study type**	**Definition of fetal growth**	**Participants**	**Neurology/neuromotor function**	**Cognitive/behavior Outcome measure**	**Cognitive/behavior findings**	**Conclusion**
Franz (58)	Single center study (Level 3 Neonatal Unit)	IUGR defined as BW < −2 SD according to British Growth Reference (59) expressed as SDS	Born < 30 wGA and BW <1,500 g BY 1996–1999 *N* = 297 preterms admitted to neonatal unit Age at assessment: 5.4 y Those with neuromotor and sensory impairment included	Neurological examination: normal, mildly abnormal (minor neurologic signs such as broad gait, dysdiadochokinesis, or dysmetria), severely abnormal (any paresis with or without spasticity, cerebral nerve palsy, or ataxia) GMFCS for level of motor function *Findings:* Neuromotor outcome associated with post-natal growth, not IUGR	K-ABC (Mental Processing Composite, MPC) Analyses adjusted for GA, sex, multiple birth, ICH > Grade 3; PVL, ROP > Grade 3, ventilation > 7 days, language, maternal education	MPC associated with IUGR (BW), early neonatal weight gain, head growth post-discharge	Intrauterine growth (weight) and in-hospital weight gain predictor of cognitive outcome Post-natal weight gain but not IUGR associated with CP HC growth but not weight gain from discharge to follow-up predictor of cognitive outcome Combined contribution of IVH and prolonged mechanical ventilation greater than the combined contribution of growth
Guellec (7)	Population based; EPIPAGE study, 9 regions in France	*N* = 2,846 SGA = <10th centile (9.2%) Mildly SGA (MSGA) = 10th– <20th centile (9.6%) AGA = >20th centile Neonatal internal reference to approach *in utero* growth restriction, similar to Mamelle et al. (60) 2 preterm groups: <28 weeks GA (*n* = 828) 28–32 weeks GA (*n* = 2018) Term born (39/40 wGA) reference group (666 children) included at birth in the same regions	All live borns 24–32 weeks GA, born 1997 in 9 regions in France *n =* 2,846 Age at assessment: 5 y: 77% neurology; 65% cognitive assessment;71% behavior questionnaires 8 y: 61%	Categorized into presence of absence of cerebral palsy (SCPE criteria for CP used) *Findings:* **29-32 weeks GA group:** Cerebral Palsy: AGA 7.7%; MSGA 4.6%; SGA 3.2% **<28 weeks GA group:** Cerebral Palsy: AGA 14%; MSGA 11.1%; SGA 18.2%	**At age 5 years**: K-ABC: Mental processing composite score (IQ equivalent). Moderate cognitive deficiency: score between 70 and 84; severe cognitive deficiency: score <70 Behavioral problems: SDQ: inattention-hyperactivity, conduct, emotional, peer problems; total behavioral difficulties score **At age 8 years**: School questionnaire (school difficulties: special schooling institution or special school, special class in mainstream school, mainstream class) or low grades Analyses adjusted for sex, GA	**29–32 weeks GA group:** Cognitive deficit: AGA 28.7%; MSGA 41.8%; SGA 40.6% Behavioral problems: AGA 19.4%; MSGA 15.7%; SGA 23.5% School difficulties: AGA 18.4%; MSGA 32.18%; SGA 28 % **<28 weeks GA group:** Cognitive deficit: AGA 38.3%; MSGA 32.1%; SGA 37.5% Behavioral problems: AGA 23.7%; MSGA 27.3%; SGA 33.3% School difficulties: AGA 33.2%; MSGA 44.8%; SGA 35.3% Analyses controlled for socio-economic factors and sex	Growth restriction associated with adverse neurodevelopmental outcomes only in the 29- to 32-week GA group; both SGA and MSGA associated with an increase of cognitive deficiency, behavioral problems, and school difficulties. CP not associated with SGA
Guellec (61)	Population based; EPIPAGE study, 9 regions in France	4 categories: Symmetric growth restriction (SGR): HC and BW <20th centile and in the same percentile range; asymmetric growth restriction (AGR): at least 1 of HC and BW <20th centile and the other in a higher decile range. Two forms of AGR: head growth restriction (HGR), weight growth restriction (WGR). AGA: both BW and HC >20th centile	All live borns 26–32 wGA BY 1997 Age at assessment: 5 y (school performance at 8 y)	Categorized into presence of absence of cerebral palsy (SCPE criteria for CP used) *Findings:* No difference between groups for CP	K-ABC: Mental processing composite score (IQ equivalent), Moderate cognitive deficiency: score between 70 and 84; severe cognitive deficiency: score <70 Behavioral problems: SDQ: inattention-hyperactivity, conduct, emotional, peer problems; total behavioral difficulties score	SGR: higher risks of both moderate and severe cognitive deficiency. HGR: only higher risk for severe cognitive deficiency No difference between groups for behavior SGR: higher rate of school difficulties than AGA children Discussion	SGR associated with impaired cognitive and school performance Outcome of AGR differed according to HC: HGR associated with impaired cognitive function; WGR not associated with cognitive outcome No higher risk for CP in SGR
Kallankari (62)	Population based (regional cohort, Finland)	FGR defined as BW < −2 SD from mean of gestation-adjusted birth weight; documented FGR due to placental insufficiency by Doppler ultrasound and histological placental perfusion defect and lack of congenital infections or malformations	Born <32 wGA (*n* = 154); term born controls (*n* = 90) BY 1998–2002 Age at assessment: 9 y *n =* 77 preterms; *n* = 18 (23% FGR), *n =* 27 term controls Only preterms without CP and without cognitive impairment included	N/A	14 subtests from NEPSY II 6 subtests from WISC-III Mean scores for 5 domains calculated: visuospatial–sensorimotor processing, attention–executive functions, language, memory–learning and social perception Analyses controlled for GA, sex, maternal education	FGR only identified risk factor for impairment in language and memory learning skills in the preterm group; no difference between preterm AGA and FGR group for attention, executive functions, visuo-spatial, sensorimotor function	FGR independently predicts poor language, memory and learning skills
Kan (63)	Population based; Victorian Infant Collaborative Study	IUGR defined as BW < −2 SD according to British Growth Reference (59); expresses as Z-score	Born <28 wGA BY 1991–1992 *n =* 179 (2.2% IUGR); Age at assessment: 8 y Those with neurosensory impairment excluded	M-ABC 2 for assessment of motor skills (dexterity, ball skills, balance) *Findings:* HC or BW, or weight catch up in early childhood not associated with outcome, but, HC age 2 y and age 8 y associated with motor outcome	WISC-III for IQ WRAT-II for educational skills (reading, spelling, arithmetic) Analyses adjusted for potential biological and environmental risk factors (IVH grade 3 or 4, cystic PVL, surgery, post-natal steroids; maternal education, social class, other languages than English)	IUGR not associated with outcome at age 8 years HC or weight at birth, or weight catch up in early childhood not associated with outcome, but HC age 2 y and age 8 y associated with cognitive outcome	Intrauterine growth mostly unrelated to cognitive and other outcomes in preterm children without neurosensory impairment, but events between post-birth and age 2 years have an effect on brain growth and function, over and above effects of prematurity
Korzeniewski (64)	Multicenter, prospective, observational study (Extremely Low Gestational Age Newborns, ELGAN)	3 groups according to BW *z*-score (65) Severe FGR: <−2, Less severe FGR: ≥−2 and <−1 No FGR: ≥−1	Born <28 w GA *N =* 889 (93%) of original cohort BY 2002–2004 *n =* 52 severe FGR *n =* 113 less severe FGR Age at assessment: 10 y	GMFCS for gross motor function MACS for hand function *Findings:* FGR not associated with poorer motor function	DAS-II, NEPSY-II for general and specific cognitive functions (including executive function, memory, attention, verbal reasoning, visuo-motor precision etc.) WIAT-III for academic achievements (reading, spelling, numerical operations) OWLS for language (oral and written), Communication Function Classification Scale, and CCC-2 for communication skills SRS for social abilities, SCQ for screen for autism; ADOS-2 for autism diagnosis Analyses adjusted for sex and racial identity	Both severe and less severe FGR similar in having lower scores than no FGR peers in verbal reasoning, listening, comprehension, visuomotor precision, word reading, working memory, pseudoword decoding, and spelling subtests; more problems with social awareness and social cognition Severe FGR group lower scores than less severe FGR group for measures of auditory attention and response as well as inhibition switching, inhibition naming, as well as having higher scores on autism screen, and a variety of communication problems	Severe FGR poses increased risk of multiple cognitive and behavioral dysfunctions There is a positive relationship between severity of FGR and severity of cognitive impairment and behavior FGR not associated with poorer motor function
Morsing (66)	Single center study, Sweden	BW < −2 SD from mean of Swedish growth standards (53)	46 fetuses with IUGR and ARED umbilical artery blood flow; delivered <30 wGA at level III perinatal center BY 1998–2004 Follow-up: *n =* 34 preterm SGA with IUGR, ARED on umbilical arterial flow antenatally *n =* 34 preterm AGA (matched for sex, GA, BY) *n =* 34 term AGA Age at assessment: 5–8 y Those with CP not excluded	Information on diagnosis of CP and GMFCS level, hearing and visual function collected at time of cognitive assessment *Findings:* No difference in CP frequency	WPPSI-III; WISC-III (IQ) SDQ, Brown ADD Scale (total scores) for socio-emotional functioning and attention difficulties Analyses controlled for parental education, neonatal septicaemia, CLD, ICH>grade 3, PVL, post-natal steroids, ROP grade 3–5	FSIQ and VIQ lower in SGA/IUGR preterms than AGA preterms; PIQ not different; this was driven by poorer performance of male preterm SGA/IUGR children No difference in SDQ and ADD scores	Male preterm SGA children with IUGR and ARED blood flow *in utero* have poorer cognitive outcome than female preterms with IUGR and AERD No higher risk for CP
Raz (67)	Single center study	IUGR defined as BW <10th centile; Z-score computed as the deviation of BW from the mean BW gestational age group, at delivery	Born 23–34+6 wGA (mean 28.6) BY 1991–2000 *n =* 25 IUGR *n =* 118 AG (appropriate growth) Age at assessment: 3–6 y Those with CP and ICH excluded	PDMS-2 for gross and fine motor skills *Findings:* Difference in gross and fine motor scores with IUGR having poorer motor skills	WPPSI-R for IQ scores PLS-3 for language skills (auditory comprehension and expressive communication) Analyses adjusted for sex, no significant difference in socio-economic class between groups	Full Scale IQ poorer in IUGR group; driven by difference in Performance IQ scores	Association between intrauterine growth and cognitive and motor outcome even within the population of preterm children who had adequate standardized birth weight
Tanis (44)	Population based regional cohort—subgroup (Longitudinal Preterm Outcome Project study), Netherlands	BW < −1 SD and birth weight > −1 SD according to GA; Dutch Kloosterman curve (45). Cut-off for SGA (> 1 SD, below the 16th percentile) according to Etude Epidemiologique sur les Petits Ages Gestationnels study	Born 31–41 wGA. AGA = 336; 216/336 moderately preterm SGA = 42; 216 32/42 moderately preterm BY 2002–2003 Age at assessment: 7 y	M-ABC 2 for assessment of motor skills (dexterity, ball skills, balance) *Findings:* No significant difference between groups	WISC III (Full Scale IQ) TEA-Ch: selective attention and Attention control RAVLT: Verbal memory NEPSY-II: Visuomotor integration BRIEF: Executive functioning in daily life Analyses adjusted for GA and sex	SGA children poorer attentional control, irrespective of GA No significant difference in other outcome measures	SGA children higher risk for difficulties with attentional control, irrespective of GA at birth. General cognitive, executive function, and motor skills not different between AGA and SGA

*AGA, appropriate for gestational age; ARED, absent or reversed end-diastolic blood flow; AGR, asymmetric growth restriction; BW, birth weight; CLD, chronic lung disease; FGR, fetal growth restriction; GA, gestational age; HGR, head growth restriction; ICH, intracranial hemorrhage; IUGR, intrauterine growth restriction; PVL, periventricular leukomalacia; ROP, retinopathy of prematurity; SGA, small for gestational age; SGR, symmetric growth restriction; w, week; WGR, weight growth restriction; y, year. ADOS, Autism Diagnostic Observation Schedule; CCL, Children's Communication Scale; CP, Cerebral Palsy; CSI, Child Symptom Inventory; Brown ADD, Brown Attention-Deficit Disorder Scales; BRIEF, Behavior Rating Inventory of Executive Function; CBCL, Child Behavior Checklist;; DAS, Differential Ability Scales; GMFCS, Gross Motor Function Classification Scale; K-ABC, Kaufmann Assessment Battery for Children; MACS, Manual Ability Classification Scale; M-ABC, Movement ABC for Children; MPC, Mental Processing Composite; NEPSY, A Developmental NEuroPSYchological Assessment; OWLS, Oral and Written Language Scales; PDMS, Peabody Developmental Motor Scales; PLS, Preschool Language Scale; RAVLT, Rey's Auditory Verbal Learning Test; SCQ, Social Communication Questionnaire; SDQ, Strengths and Difficulties Questionnaire; TeaCH, Test of Everyday Attention in Children; WIAT, Wechsler Individual Achievement Test; WISC, Wechsler Intelligence Scales for Children; WPPSI, Wechsler Preschool and Primary Scale of Intelligence; WRAT, Wide Range Achievement Test. The terms SGA, IUGR, FGR were used as in the study that is described*.

### Neuromotor Outcomes

An association between SGA and/or FGR with CP in children born preterm has been reported from CP registers, for example, from the Surveillance of Cerebral Palsy in Europe (SCPE), who found that those born with BW for GA below the 10th percentile (using fetal growth standards) had a higher risk for CP than those with a BW between 25th and 75th centile. This was found for the GA range of 32–41 weeks and was similar for all CP subtypes. The association between SGA at birth and CP, however, was not as clear for those born very or extremely preterm. Interestingly, a higher risk is also reported for those with BW above the 97th centile (11). Similar data are reported from other cohorts. For example, Jacobsson (49), using data from the CP register for West Sweden, after adjustment for maternal height, weight in early pregnancy, parity, ethnic origin and baby's sex, did not find an increased proportion of children who were SGA at birth in the preterm group (born <36+6 wGA), whereas in the term born group children with CP were significantly more likely to have been SGA at birth. It should be kept in mind that these are associations and that this does not necessarily mean that growth restriction causes CP. For very and extremely preterm children, the evidence suggests that other biological risk factors, in particular, focal preterm brain injury, pose a higher risk for severe neuromotor impairment than growth restriction. The findings of the studies reviewed here (Table 2) do not suggest that SGA and/or FGR pose a significant independent risk for CP in children born preterm, and this appears to be the case across the preterm gestational age range (7, 49, 58, 61, 66). Post-natal growth appears to be an important factor as, for example, indicated by the study by Franz et al. (58) in school aged children born <30 wGA and BW <1,500 g, who found that neuromotor outcome was associated with post-natal growth but not FGR, and this was still the case once analyses had been adjusted for important other biological risk factors including brain injury.

Neuromotor function in the absence of CP has been reported by several groups. The studies reviewed here report contradictory findings. Tanis (44), examined motor skills with the M-ABC in school aged children born 31–41 weeks of gestation in a population based cohort in the Netherlands, and did not find a difference in motor skills for the moderately preterm group between those who were AGA and those who were SGA at birth. Korzeniewski et al. (64), reported for the extremely preterm born ELGAN cohort no differences in motor function (assessed with the GMFC and MACS) at school age between those born after FGR and those without FGR. In another study in extremely preterm children Kan et al. (63), report M-ABC data from the Victorian Infant Collaborative study, a population based study of children born extremely preterm, and did not find an association between motor skills and FGR. However, they did see a significant association between head circumference at age 2 years and age 8 years with motor skills, suggesting that post-natal and post-neonatal brain growth is a crucial factor for motor outcome at school age, independently of presence of absence of FGR. In contrast to the findings by Kan (63), Tanis, and Korzeniewski (44, 63, 64) from 3 large population based cohorts, Raz et al. (67), report for a single center study of children born <34+6 wGA, using the PDMS-2, poorer gross and fine motor skills as well as performance IQ scores, for those with FGR compared to those born with appropriate for GA birth weight, and, importantly, an association between intrauterine growth and motor skills as well as Performance IQ even in those who had adequate standardized birth weight. Despite these inconsistent findings between studies, it appears to emerge that post-natal and post-neonatal brain growth is more important than intrauterine growth for neuromotor development in those who do not develop CP, which is also supported by Neubauer et al. (68), who examined 448 children born <32 w GA (19.9% SGA), and found that head size at age 3 months was the best predictor for motor (and cognitive) outcome at age 24 months. Visuo-motor skills at age 11 years were examined in the study by Kok et al. (69), for a cohort of preterm children (born <33 wGA) born FGR and who had antenatal Doppler studies performed; comparison was made between the group with and without “brain sparing.” No significant differences were see in any of the tests (Beery-Buktencia Developmental test of Visuo-motor Integration, Motor Accuracy Test (MAT), Motor-Free Visual Perception Test (MVPT-R), and the balls skills subtest from the M-ABC).

### Cognitive and Behavioral Outcomes

It is well-known that prematurity, in particular, very and extremely preterm birth, poses a risk for impairment of general cognitive abilities as well as difficulties in specific cognitive functions such as memory functions, processing speed, cognitive flexibility, attentional abilities, as well as socio-emotional behavior, all of which will affect for academic progress and peer relationships. Being born preterm and SGA and/or having FGR may be an independent risk factor for impairment of long term cognitive and behavioral outcomes, over and above other relevant biological and social risk factors. Long term outcomes of preterm birth are affected by a number of factors and it is often complicated to establish an independent effect of SGA and/or FGR on long term cognitive outcomes in the context of prematurity. Often, those who are SGA and/or have had FGR, also are those who, compared to those born AGA, have more other neonatal morbidities that have been show to affect outcome (such as chronic lung disease, neonatal sepsis, etc.), which has been illustrated, for example, by the large EPIPAGE study (61). In addition, often, there are no detailed fetal growth measures or information on fetal Doppler studies available, which can make it difficult to establish the exact cause of a preterm infant's SGA birth weight. Direct comparison of study findings is difficult since slightly different criteria for definition of SGA and/or FGR are used in different studies. In addition, although most studies have assessed general cognitive abilities with either the Wechsler Intelligence Scales (44, 62, 63, 66, 67), the Kaufmann ABC (7, 58, 61), or the NEPSY assessment (62, 64), different studies focused on different specific cognitive functions, and there is a large variety of different tests used. Furthermore, in some studies participants with neurosensory impairment were not excluded from analyses on cognitive and behavioral function, which complicates comparison with those studies in which only those without neurosensory impairment were assessed. However, overall, the studies reviewed here suggest that preterm children born SGA and/or having had FGR, compared to their AGA preterm peers, are at increased risk of developing cognitive and behavioral difficulties, although some study findings are inconsistent. With the exception of the study on the Victorian Infant Collaborative Study cohort (63), who did not find an association between FGR and cognitive abilities at school age, but between post-neonatal head growth and cognitive development, the large multicenter or population based studies reviewed here (7, 44, 61, 64) report an effect of FGR (it has to be kept in mind that in most studies SGA was used as a surrogate marker for FGR) on long term cognitive function, and, in the majority of the studies, this was still the case once other biological and social risk factors are considered. Guellec (7), reported school age outcome data for the EPIPAGE cohort (GA range 24–32 weeks) and divided the SGA participants into 2 groups, mildly SGA and SGA, and also formed 2 GA groups (<28 wGA, 29–32 wGA). They found impaired cognitive function, behavioral problems, and school difficulties at school age were more frequent in both the mildly SGA and the SGA born children in the 29–32 wGA group but not in the more immature GA group.

Korzeniewski et al. (64), examined school age outcomes for the extremely preterm (<28 wGA) born ELGAN cohort, focusing on general and specific cognitive abilities (including executive function, memory, attention, verbal reasoning, visuo-motor precision), communication abilities, and socio-emotional behavior. They did find overall a positive relationship between severity of FGR and severity of cognitive impairment and behavior for most areas assessed, and, in addition, that those with the most severe FGR also had more problems than those with less severe FGR in auditory attention, inhibition switching, inhibition naming, higher scores on autism screen, and a variety of communication problems. Kallankari et al. (62), followed a Finnish cohort of children born at a more mature GA (<32 wGA) for whom FGR due to placental insufficiency was documented by Doppler ultrasound and histological placental perfusion defect. They reported that FGR independently predicted poor language, memory and learning skills, but did not find an effect of FGR on attention, executive function, visuo-spatial, or sensorimotor function. For a cohort with a larger range of GA at birth (31–41 wGA), Tanis et al. (44), established that those born SGA were at higher risk for difficulties with attentional control, irrespective of GA at birth, but that there was no difference to those born AGA for measures of general cognitive abilities, executive function, visuomotor integration, or memory function. These findings on attention difficulties in children born SGA and/or FGR are consistent with those from a large nationwide case-control study in Finland that investigated associations between prematurity and fetal growth in relation to Attention Deficit Hyperactivity Disorder (ADHD) (70). This study found that prematurity was a risk factor for ADHD across the preterm gestational age range, and that with lowest weight for GA (< −2 SD) had the highest risk for ADHD, together with those being more than 2 SD large for GA.

Some studies have investigated childhood outcomes of preterm children born SGA and/or FGR for whom information on umbilical artery blood flow was available. Morsing et al. (66), examined outcome in a cohort of fetuses with FGR and absent or reversed end-diastolic umbilical artery (AERD) blood flow who were delivered before 30 wGA, and compared them to a control group of preterm infants of the same GA born with AGA birth weight. At school age those with AERD had poorer full scale and verbal IQ than those born AGA and this difference was driven by the poor performance of male participants in the FGR/AERD group. These findings are inconsistent with those of Valcamonico et al. (71), who found a higher incidence of severe neurological problems (CP, visual and/or hearing impairment) but no difference in general cognitive abilities in a group of very preterm born (mean GA at birth 33.3 weeks; 35 % SGA with BW <5th centile) school aged children who had AERD when compared to a group of children (mean GA at birth 30.8 weeks; 38% SGA) who did not have AERD. It is possible that the difference in outcomes between these studies are explained by the different proportions of children having been SGA at birth, despite all having had AERD; furthermore there are differences between studies in the methodology of the Doppler studies and definition of SGA and/or fetal growth. van den Broek et al. (72), compared behavior in a large cohort of 11 years old children born preterm (<33 wGA) in whom weekly antenatal Doppler studies were done to assess the ratio between umbilical and cerebral pulsatility index (U/C ration) for assessment of “brain sparing.” In this study, rather than using centile curves, fetal growth ratio was defined as the ratio of the observed birth weight to the expected mean birth weight for gestational age, based on Dutch intrauterine growth curves. At age 5 years significant difference in the proportion of those with Full Scale IQ <85 in the group with “brain sparing,” but no significant difference for mean FS IQ between the groups. At age 11 years no significant association was seen between parent and teacher rating of behavior on the CBCL and U/C ratio after controlling for neonatal variables and IQ at age 5 years, indicating that “brain sparing” was not an independent risk factor for behavioral difficulties at age 11 y.

Most of the existing studies in term born babies use growth or birth weight <10th centile, often based on different growth standards, as definition of FGR. Using more stringent FGR bands may be useful in future work in order to examine in more detail whether there is an effect of only severe FGR on development. Kan et al. (63) studied long term cognitive outcome in extremely preterm born children, using stricter criteria for FGR (< −2 SD) and did not find an association between FGR and outcome, but strong associations between post-natal head growth and outcome, which emphasizes the importance of head growth, very likely independently of the severity of FGR. The importance of post-neonatal head growth for cognitive development in children born SGA and/or FGR has also been highlighted by the study by Franz et al. (58), who reported for a single center cohort born <30 wGA that FGR, in-hospital weight gain, and post-neonatal head growth (but not weight gain) predicted general cognitive abilities at school age (63).

## Conclusions

Overall, existing studies are heterogeneous, for example, with regards to definitions of growth restriction and small for gestational age, time of onset of FGR, inclusion and exclusion criteria, outcome measures, all of which makes comparison between studies difficult.

However, in summary, the literature reviewed here suggests that cognitive development in term born children is not hugely impacted by them being born SGA or FGR, particularly in the case of general intellectual function, on which the majority of studies have been conducted. There is some limited evidence that term born growth restricted children may experience problems with attention, and such difficulties may have more impact on children's learning and behavior as they progress through school, suggesting that this would be a useful area for further work. The risk for developing CP is higher in children born SGA with FGR; currently there is contradictory information available on neuromotor function in the absence of CP.

In contrast, preterm born SGA and/or FGR children are at great risk of poorer general intellectual functioning than their AGA counterparts. These children are also at risk for difficulties with attentional control, and some studies suggest that memory, executive function, and communication skills are also affected. It does not appear that SGA and/or FGR *per se* pose a higher risk for CP; information on motor outcome in the absence of CP is, similar to the term born children, somewhat contradictory.

Post-neonatal head growth and head growth in infancy and early childhood emerges as an important factor for long term neurodevelopmental outcome.

There is some inconsistency between the conclusions of our review of school aged children and those studies that report follow up data acquired in the toddler years. This could result from certain cognitive difficulties taking time to emerge and/or these being more subtle problems that are nevertheless clinically and functionally important. It is important to continue to follow up children into the school age years because these difficulties may take time to emerge, and may be more visible in the more demanding school environment.

## Author Contributions

BV and CE have made equal contributions to this work. Both provide approval for publication of the work and agree to be accountable for all aspects of the work.

### Conflict of Interest Statement

The authors declare that the research was conducted in the absence of any commercial or financial relationships that could be construed as a potential conflict of interest.

## Abbreviations

AGA: appropriate for gestational age
ANT: Amsterdam Neuropsychological Tasks
AGR: asymmetric growth restriction
ARED: absent or reversed end-diastolic blood flow
BW: Birth weight
BL: Birth Length
BRIEF: Behavior Rating Inventory of Executive Function
CLD: Chronic lung disease
CP: Cerebral Palsy
FGR: Fetal growth restriction
FSIQ: Full Scale IQ Score
GA: Gestational age
HC: Head Circumference
HGR: Head growth restriction
ICH: Intracranial hemorrhage
IQ: Intelligence Quotient Score
IUGR: Intrauterine growth restriction
K-ABC: Kaufmann Assessment Battery for Children
M-ABC: Movement ABC for Children
MHPT: Moray House Picture Test
NEPSY: A Developmental Neuropsychological Assessment
PIQ: Performance IQ Score
POBW: Percentage of optimal birth weight
RAVLT: Rey's Auditory Verbal Learning Test
ROYCF: Rey Osterreith Complex Figure test
SES: Socio-economic status
SGA: Small for gestational age
TeaCH: Test of Everyday Attention in Children
VIQ: Verbal IQ score
WASI: Wechsler Abbreviated Scales of Intelligence
w: Week
WGR: Weight growth restriction
WISC: Wechsler Intelligence Scales for Children
WISC-R: Wechsler Intelligence Scale for Children – revised
WPPSI: Wechsler Preschool and Primary Scale of Intelligence
WRAT: Wide Range Achievement Test
y: Year.

## References

[1] Murray E Fernandes M Fazel M Kennedy SH Villar J Stein A. Differential effect of intrauterine growth restriction on childhood neurodevelopment: a systematic review. BJOG An Int J Obstet Gynaecol. (2015) 122:1062–72. 10.1111/1471-0528.13435 25990812

[2] van Wassenaer A. Neurodevelopmental consequences of being born SGA. Pediatr Endocrinol Rev. (2005) 2:372–7. 16429113

[3] Maulik D. Fetal growth compromise: definitions, standards, and classification. Clin Obs Gynecol. (2006) 49:214–8. 10.1097/00003081-200606000-00004 16721101

[4] Baschat A. Neurodevelopment after fetal growth restriction. Fetal Diagn Ther. (2014) 36:136–42. 10.1159/000353631 23886893

[5] Goldenberg RL Culhane JF Iams JD Romero R. Epidemiology and causes of preterm birth. Lancet. (2008) 371:75–84. 10.1016/S0140-6736(08)60074-4 18177778 PMC7134569

[6] Walker D Marlow N. Neurocognitive outcome following fetal growth restriction. Arch Dis Child Fetal Neonatal Ed. (2008) 93:F322–5. 10.1136/adc.2007.120485 18381841

[7] Guellec I Lapillonne A Renolleau S Charlaluk M Roze J Marret S Neurologic outcomes at school age in very preterm infants born with severe or mild growth restriction. Pediatrics. (2011) 127:e883–91. 10.1542/peds.2010-2442 21382951

[8] Lundgren EM Cnattingius S Jonsson B Tuvemo T. Birth characteristics and different dimensions of intellectual performance in young males: a nationwide population-based study. Acta Paediatr Int J Paediatr. (2003) 92:1138–43. 10.1111/j.1651-2227.2003.tb02473.x 14632327

[9] Leonard H Nassar N Bourke J Blair E Mulroy S De Klerk N. Relation between intrauterine growth and subsequent intellectual disability in a ten-year population cohort of children in Western Australia. Am J Epidemiol. (2008) 167:103–11. 10.1093/aje/kwm245 17898000

[10] Freire G Shevell M Oskoui M. Cerebral palsy: phenotypes and risk factors in term singletons born small for gestational age. Eur J Paediatr Neurol. (2015) 19:218–25. 10.1016/j.ejpn.2014.12.005 25596065

[11] Jarvis S Glinianaia S Torrioli M Platt M Miceli M Jouk P Surveillance of cerebral palsy in Europe (SCPE) collaboration of European cerebral palsy registers. Cerebral palsy and intrauterine growth in single births: European collaborative study. Lancet. (2003) 4:1106–11. 10.1016/S0140-6736(03)14466-2 14550698

[12] Palisano R Rosenbaum P Walter S Russell D Wood E Galuppi B. Development and reliability of a system to classify gross motor function in children with cerebral palsy. Dev Med Child Neurol. (1997) 39:214–23. 10.1111/j.1469-8749.1997.tb07414.x 9183258

[13] Eliasson A Krumlinde-Sundholm L Rösblad B Beckung E Arner M Ohrvall A. The manual ability classification system (MACS) for children with cerebral palsy: scale development and evidence of validity and reliability. Dev Med Child Neurol. (2006) 48:549–54. 10.1017/S0012162206001162 16780622

[14] Henderson S Sugden D Barnett A Movement Assessment Battery for Children—Second Edition (MABC-2). London: Psychological Corporation (2007).

[15] Folio M Fewell R Peabody Developmental Motor Scales-Second Edition. Austin, TX: Pro-Ed, Inc.

[16] Wechsler D The Wechsler Intelligence Scale for Children. 5th edn. Bloomington, MN: Pearson (2014).

[17] Wechsler D Wechsler Abbreviated Scale of Intelligence. San Antonio, TX: Psychological Corporation (1999).

[18] Wechsler D Wechsler Preschool and Primary Scale of Intelligence - Fourth Edition (WPPSITM - IV). Bloomington, MN: Pearson, Psychological Corporation (2012).

[19] Kauffman A Kauffman N Assessment Battery for Children (K-ABC) Hebrew Version. Jerusalam: Ministry of Education (1996).

[20] Manly T Anderson V Nimmo-Smith I Turner A Watson P Robertson I. The differential assessment of children's attention: the test of everyday attention for children (TEA-Ch), normative sample and ADHD performance. J Child Psychol Psychiatry. (2001) 42:1065–81. 10.1111/1469-7610.00806 11806689

[21] Vakil E Blachstein H Rochberg J Vardi M Characterization of memory impairment following closed-head injury in children using the rey auditory verbal learning test (AVLT) child neuropsychology. Neuropsychol Dev Cogn Sect C. (2004) 10:57–66. 10.1080/09297040490911078 15590485

[22] Osterreith P Le test do copie d'une figure complex. Arch Psychol. (1944) 30:206–36.

[23] Korkman M Kirk U Kemp S NEPSY-II: Clinical and Interpretive Manual. San Antonio, TX: Pearson Education (2007).

[24] Gioia G Isquith PK Guy SC Kenworthy L Behavior Rating Inventory of Executive Function. Odessa, FL: Psychological Assessment Resources (2000).

[25] Goodman R. The strengths and difficulties questionnaire: a research note. J Child Psychol Psychiatry. (1997) 38:581–6. 10.1111/j.1469-7610.1997.tb01545.x 9255702

[26] Constantino J Gruber C Social Responsiveness Scale (SRS2). Los Angeles, CA: Western Psychological Services (2012).

[27] Rutter M Bailey A Lord C The Social Communication Questionnaire. Los Angeles, CA: Western Psychological Services (2003).

[28] Lord C Rutter M DiLavore P Risi S Gotham K Bishop S Autism Diagnostic Observation Schedule. 2nd ed. Torrance, CA: Western Psychological Services (2012).

[29] Beukers F Aarnoudse-Moens CSH van Weissenbruch MM Ganzevoort W van Goudoever JB van Wassenaer-Leemhuis AG. Fetal growth restriction with brain sparing: neurocognitive and behavioral outcomes at 12 years of age. J Pediatr. (2017) 188:103–9.e2. 10.1016/j.jpeds.2017.06.003 28693788

[30] Gardosi J Mongelli M Wilcox M Change A. An adjustable fetal weight standard. Ultrasound Obstet Gynecol. (1995) 6:167–74. 10.1046/j.1469-0705.1995.06030168.x 8521065

[31] Emond A Lira P Lima M Grantham-McGregor SM Ashworth A. Development and behaviour of low-birthweight term infants at 8 years in northeast Brazil: a longitudinal study. Acta Paediatr. (2006) 95:1249–57. 10.1080/08035250600615127 16982498

[32] Fattal-Valevski A Toledano-Alhadef H Leitner Y Geva R Eshel R Harel S. Growth patterns in children with intrauterine growth retardation and their correlation to neurocognitive development. J Child Neurol. (2009) 24:846–51. 10.1177/0883073808331082 19617460

[33] Lieberman J Fraser D Weitzman S Glezerman M Birthweight curves in southern Israel populations. Isr J Med Sci. (1993) 5:198–203. 8155098

[34] Gale CR O'Callaghan FJ Godfrey KM Law CM Martyn CN. Critical periods of brain growth and cognitive function in children. Brain. (2004) 127:321–9. 10.1093/brain/awh034 14645144

[35] Blair E Liu Y de Klerk N. Optimal fetal growth for the Caucasian singleton and assessment of appropriateness of fetal growth: an analysis of a total population perinatal database. BMC Pediatr. (2005) 5:13. 10.1186/1471-2431-5-13 15910694 PMC1174874

[36] Gale CR O'Callaghan FJ Bredow M Martyn CN. The influence of head growth in fetal life, infancy, and childhood on intelligence at the ages of 4 and 8 years. Pediatrics. (2006) 118:1486–92. 10.1542/peds.2005-2629 17015539

[37] Geva R Eshel R Leitner Y Fattal-Valevski A Harel S. Memory functions of children born with asymmetric intrauterine growth restriction. Brain Res. (2006) 1117:186–94. 10.1016/j.brainres.2006.08.004 16962082

[38] Geva R Eshel R Leitner Y Fattal-Valevski A Harel S. Verbal short-term memory span in children: long-term modality dependent effects of intrauterine growth restriction. J Child Psychol Psychiatry. (2008) 49:1321–30. 10.1111/j.1469-7610.2008.01917.x 19120711

[39] Lawlor DA. Intrauterine growth and intelligence within sibling pairs: findings from the aberdeen children of the 1950s cohort. Pediatrics. (2006) 117:e894–902. 10.1542/peds.2005-2412 16651293

[40] Leitner Y Fattal-valevski A Geva R Eshel R Toledano-alhadef H Rotstein M. Neurodevelopmental outcome of children a longitudinal, 10-year prospective study. J Child Neurol. (2009) 22:580–7. 10.1177/0883073807302605 17690065

[41] Niklasson A Erison A Fryer J Karlberg J Lawrence C Karlberg P. An update of the Swedish reference standards for weigh, length and head circumference at birth for given gestational age. Acta Paediatr Scan. (1991) 80:756–62. 10.1111/j.1651-2227.1991.tb11945.x 1957592

[42] Sommerfelt K Sonnander K Skranes J Andersson H Ahlsten G Ellertsen B. Neuropsychologic and motor function in small-for-gestation preschoolers. Pediatr Neurol. (2002) 26:186–91. 10.1016/S0887-8994(01)00381-2 11955924

[43] Bakketeig L. Current growth standards, definitions, diag- nosis and classification of fetal growth retardation. Eur J Clin Nutr. (1998) 52:1–4. 9511013

[44] Tanis JC Van Braeckel KNJA Kerstjens JM Bocca-Tjeertes IFA Reijneveld SA Bos AF. Functional outcomes at age 7 years of moderate preterm and full term children born small for gestational age. J Pediatr. (2015) 166:552–8. 10.1016/j.jpeds.2014.11.043 25575420

[45] Kloosterman G On intrauterine growth: the significance of prenatal care. Int J Gynaecol Obstet. (1970) 8:895–912. 10.1002/j.1879-3479.1970.tb00313.x

[46] Theodore RF Thompson JMD Waldie KE Becroft DMO Robinson E Wild CJ. Determinants of cognitive ability at 7 years: a longitudinal case-control study of children born small-for-gestational age at term. Eur J Pediatr. (2009) 168:1217–24. 10.1007/s00431-008-0913-9 19165501

[47] Thompson J Mitchell E Borman B Sex specific birthweight percentiles by gestational age for New Zealand. N Z Med J. (1994) 10:1–3. 8295745

[48] Blair EM Nelson KB. Fetal growth restriction and risk of cerebral palsy in singletons born after at least 35 weeks' gestation. Am J Obstet Gynecol. (2015) 212:520.e1–7. 10.1016/j.ajog.2014.10.1103 25448521

[49] Jacobsson B Ahlin K Francis A Hagberg G Hagberg H Gardosi J. Cerebral palsy and restricted growth status at birth: population-based case-control study. BJOG An Int J Obstet Gynaecol. (2008) 115:1250–5. 10.1111/j.1471-0528.2008.01827.x 18715410

[50] Drummond P Colver A. Analysis by gestational age of cerebral palsy in singleton births in north-east England 1970-94. Paediatr Perinat Epidemiol. (2002) 16:172–80. 10.1046/j.1365-3016.2002.00408.x 12060314

[51] Himmelmann K Ahlin K Jacobsson B Cans C Thorsen P. Risk factors for cerebral palsy in children born at term. Acta Obs Gynecol Scand. (2011) 90:1070–81. 10.1111/j.1600-0412.2011.01217.x 21682697

[52] Tin W Wariyar U Hey E. Selection biases invalidate current low birthweight for gestation standards. Br J Obs Gynaecol. (1997) 104:180–5. 10.1111/j.1471-0528.1997.tb11041.x 9070135

[53] Maršál K Persson P Larsen T Lilja H Selbing A Sultan B. Intrauterine growth curves based on ultrasonically estimated foetal weights. Acta Paediatr. (1996) 85:843–8. 10.1111/j.1651-2227.1996.tb14164.x 8819552

[54] Stoknes M Andersen G Elkamil A Irgens L Skranes J Salvesen K. The effects of multiple pre- and perinatal risk factors on the occurrence of cerebral palsy. A Norwegian register based study. Eur J Paediatr Neurol. (2012) 16:56–63. 10.1016/j.ejpn.2011.10.004 22104566

[55] Matte TD Bresnahan M Begg MD Susser E. Influence of variation in birth weight within normal range and within sibships on IQ at age 7 years: cohort study. BMJ. (2001) 323:310–4. 10.1136/bmj.323.7308.310 11498487 PMC37317

[56] Diamond A. Executive functions. Annu Rev Psychol. (2013) 64:135–68. 10.1146/annurev-psych-113011-143750 23020641 PMC4084861

[57] Lahti J Räikkönen K Kajantie E Heinonen K Pesonen A Järvenpää A. Small body size at birth and behavioural symptoms of ADHD in children aged five to six years. J Child Psychol Psychiatry. (2006) 47:1167–74. 10.1111/j.1469-7610.2006.01661.x 17076756

[58] Franz AR Pohlandt F Bode H Mihatsch WA Sander S Kron M. Intrauterine, early neonatal, and postdischarge growth and neurodevelopmental outcome at 5.4 years in extremely preterm infants after intensive neonatal nutritional support. Pediatrics. (2009) 123:e101–9. 10.1542/peds.2008-1352 19117831

[59] Cole T Freeman J Preece M. British 1990 growth reference centiles for weight, height, body mass index and head circumference fitted by maximum penalized likelihood. Stat Med. (1998) 17:407–29. 10.1002/(SICI)1097-0258(19980228)17:4<407::AID-SIM742>3.0.CO;2-L 9496720

[60] Mamelle N Boniol M Rivière O Joly M Mellier G Maria B. Identification of newborns with Fetal Growth Restriction (FGR) in weight and/or length based on constitutional growth potential. Eur J Pediatr. (2006) 165:717–25. 10.1007/s00431-005-0045-4 16835759

[61] Guellec I Marret S Baud O Cambonie G Lapillonne A Roze JC. Intrauterine growth restriction, head size at birth, and outcome in very preterm infants. J Pediatr. (2015) 167:975–81e2. 10.1016/j.jpeds.2015.08.025 26384436

[62] Kallankari H Kaukola T Olsén P Ojaniemi M Hallman M. Very preterm birth and foetal growth restriction are associated with specific cognitive deficits in children attending mainstream school. Acta Paediatr Int J Paediatr. (2015) 104:84–90. 10.1111/apa.12811 25272976

[63] Kan E Roberts G Anderson PJ Doyle LW. The association of growth impairment with neurodevelopmental outcome at eight years of age in very preterm children. Early Hum Dev. (2008) 84:409–16. 10.1016/j.earlhumdev.2007.11.002 18096332

[64] Korzeniewski SJ Allred EN Joseph RM Heeren T Kuban KCK O'Shea TM. Neurodevelopment at age 10 years of children born <28 weeks with fetal growth restriction. Pediatrics. (2017) 140:e20170697. 10.1542/peds.2017-0697 29030525 PMC5654396

[65] Yudkin P Aboualfa M Eyre J Redman C Wilkinson A. New birthweight and head circumference centiles for gestational ages 24 to 42 weeks. Early Hum Dev. (1987) 15:45–52. 10.1016/0378-3782(87)90099-5 3816638

[66] Morsing E Asard M Ley D Stjernqvist K Marsal K. Cognitive function after intrauterine growth restriction and very preterm birth. Pediatrics. (2011) 127:e874–82. 10.1542/peds.2010-1821 21382944

[67] Raz S Debastos AK Newman JB Batton D. Intrauterine growth and neuropsychological performance in very low birth weight preschoolers. J Int Neuropsychol Soc. (2012) 18:200–11. 10.1017/S1355617711001767 22300634

[68] Neubauer V Griesmaier E Pehböck-Walser N Pupp-Peglow U Kiechl-Kohlendorfer U. Poor postnatal head growth in very preterm infants is associated with impaired neurodevelopment outcome. Acta Paediatr. (2013) 102:883–8. 10.1111/apa.12319 23772884

[69] Kok JH Prick L Merckel E Everhard Y Verkerk GJQ Scherjon SA. Visual function at 11 years of age in preterm-born children with and without fetal brain sparing. Pediatrics. (2007) 119:e1342–50. 10.1542/peds.2005-2857 17545364

[70] Sucksdorff M Lehtonen L Chudal R Suominen A Joelsson P Gissler M. Preterm birth and poor fetal growth as risk factors of attention-deficit/hyperactivity disorder. Pediatrics. (2015) 136:e599–608. 10.1542/peds.2015-1043 26304830

[71] Valcamonico A Accorsi P Battaglia S Soregaroli M Beretta D Frusca T. Absent or reverse end-diastolic flow in the umbilical artery: intellectual development at school age. Eur J Obs Gynecol Reprod Biol. (2004) 114:23–8. 10.1016/j.ejogrb.2003.09.033 15099866

[72] van den Broek AJM Kok JH Houtzager BA Scherjon SA. Behavioural problems at the age of eleven years in preterm-born children with or without fetal brain sparing: a prospective cohort study. Early Hum Dev. (2010) 86:379–84. 10.1016/j.earlhumdev.2010.04.007 20554130

